# Supplementary material for: Atypical cognitive training-induced learning and brain plasticity and their relation to insistence on sameness in children with autism
Source: eLife. 2023 Aug 3;12:e86035. doi: 10.7554/eLife.86035 (PMC10550286; doi:10.7554/eLife.86035)
Supplement: Supplementary file 1. [file elife-86035-supp1.docx]

**Supplementary File 1**

**Table 1**. Demographic and clinical measures.

| Measure | ASD (n = 35) | TD (n = 28) | *t /*$\boldsymbol{\chi}$*^2^* | *p* |
| --- | --- | --- | --- | --- |
| Gender (M/F) | 29/6 | 22/6 | 0.19^a^ | 0.667^a^ |
| Age (years) | 9.98 ﻿± 0.92 | 10.00 ± 1.09 | -0.06 | 0.953 |
| WASI scale |  |  |  |  |
| Verbal IQ | 112.54 ﻿± 14.33 | 119.14 ± 13.01 | -1.89 | 0.063 |
| Performance IQ | 119.40 ﻿± 19.44 | 114.07 ﻿± 11.23 | 1.29 | 0.203 |
| Full IQ | 117.71 ﻿± 15.72 | 118.64 ﻿± 9.41 | -0.28 | 0.784 |
| Head motion^†^ |  |  |  |  |
| Pre-training scan |  |  |  |  |
| Transition x | 0.41 ± 0.30 | 0.40 ± 0.27 | 0.19 | 0.850 |
| Transition y | 0.68 ± 0.51 | 0.58 ± 0.41 | 0.76 | 0.450 |
| Transition z | 1.40 ± 0.70 | 1.36 ± 0.86 | 0.19 | 0.849 |
| Rotation pitch | 0.03 ± 0.01 | 0.03 ± 0.02 | 0.51 | 0.611 |
| Rotation roll | 0.01 ± 0.01 | 0.01 ± 0.01 | -0.06 | 0.953 |
| Rotation yaw | 0.01 ± 0.01 | 0.01 ± 0.01 | -1.33 | 0.191 |
| Mean frame-wise displacement | 0.11 ± 0.06 | 0.09 ± 0.04 | 1.59 | 0.119 |
| Max frame-wise displacement | 0.88 ± 0.49 | 0.71 ± 0.51 | 1.16 | 0.254 |
| Post-training scan |  |  |  |  |
| Transition x | 0.52 ± 0.40 | 0.46 ± 0.32 | 0.59 | 0.560 |
| Transition y | 0.97 ± 0.88 | 0.83 ± 0.85 | 0.56 | 0.581 |
| Transition z | 1.95 ± 1.27 | 1.35 ± 0.93 | 1.84 | 0.073 |
| Rotation pitch | 0.04 ± 0.04 | 0.04 ± 0.03 | 0.81 | 0.425 |
| Rotation roll | 0.02 ± 0.01 | 0.01 ± 0.01 | 0.92 | 0.364 |
| Rotation yaw | 0.01 ± 0.01 | 0.01 ± 0.01 | 0.52 | 0.608 |
| Mean frame-wise displacement | 0.13 ± 0.07 | 0.09 ± 0.04 | 2.22 | 0.032 |
| Max frame-wise displacement | 1.17 ± 0.73 | 0.87 ± 0.68 | 1.41 | 0.165 |
| ADI-R |  |  |  |  |
| Social | 20.09 ± 6.14 |  |  |  |
| Verbal | 16.17 ± 4.46 |  |  |  |
| Repetitive behavior | 5.43 ± 2.83 |  |  |  |
| Development | 3.17 ± 1.07 |  |  |  |
| ADOS^††^ |  |  |  |  |
| Social/Affect | 9.26 ± 3.00 |  |  |  |
| Restricted and repetitive behavior | 2.91 ± 1.48 |  |  |  |
| Severity scores | 7.18 ± 1.78 |  |  |  |
| Total | 12.18 ± 3.62 |  |  |  |
| RRIB sub-scores (based on ADI)^†††^ |  |  |  |  |
| Insistence on sameness | 1.10 ± 1.30 |  |  |  |
| Circumscribed interests | 2.62 ± 1.52 |  |  |  |
| Repetitive motor behavior | 2.75 ± 1.72 |  |  |  |

The mean, standard deviation of measure, *t* or $\chi$*^2^*, and *P* value of two sample t-test/chi-square test are shown here.

Abbreviations: ASD, children with autism spectrum disorder; TD, typically developing children; M, male; F, female; IQ, intelligence quotient; WASI, ﻿Wechsler Abbreviated Scale of Intelligence; ADI-R, Autism Diagnostic Interview-Revised (diagnostic scores); ADOS, Autism Diagnostic Observation Schedule-new algorithm; RRIB, restricted and repetitive interests and behaviors.

^a^ The statistic value were obtained using a chi-square test;

^†^ Data from fourteen children with ASD and four TD children are excluded;

^††^ Data from one participant are missing;

^†††^Data from two participants are missing.
