# Supplementary material for: Atypical cognitive training-induced learning and brain plasticity and their relation to insistence on sameness in children with autism
Source: eLife. 2023 Aug 3;12:e86035. doi: 10.7554/eLife.86035 (PMC10550286; doi:10.7554/eLife.86035)
Supplement: Supplementary file 2. [file elife-86035-supp2.docx]

**Supplementary File 2**

**Table 2**: Number of participants included in each analysis

|  | ASD | TD |
| --- | --- | --- |
| Behavioral analysis |  |  |
| Training task | 29 | 26 |
| Math verification task | 35 | 28 |
| Math production task and strategy assessment | 33 | 28 |
| Brain measures and brain-behavior analysis | 21 | 24 |
| RRIB-brain-behavior moderation analysis | 19 | n.a. |

Abbreviations: ASD, children with autism spectrum disorder; TD, typically developing children; n.a., not available.
