# Supplementary material for: Atypical cognitive training-induced learning and brain plasticity and their relation to insistence on sameness in children with autism
Source: eLife. 2023 Aug 3;12:e86035. doi: 10.7554/eLife.86035 (PMC10550286; doi:10.7554/eLife.86035)
Supplement: Supplementary file 3. [file elife-86035-supp3.docx]

**Supplementary File 3**

**Table 3:** Results of repeated measures ANOVA for behavioral performance

|  | Measure | Effect | *F* | *df* | *η^2^_p_* | BF | *p* |
| --- | --- | --- | --- | --- | --- | --- | --- |
| Training task | **IES** | Time Effect | 41.82 | 4,212 | **0.44** | **>100** | **<0.001** |
|  |  | Group Effect | 0.01 | 1,212 | <0.01 | 0.33 | 0.923 |
|  |  | Interaction | 0.89 | 4,212 | 0.02 | 0.08 | 0.471 |
|  |  | **ASD** |  |  |  |  |  |
|  |  | Time Effect | 24.27 | 4,112 | **0.46** | **>100** | **<0.001** |
|  |  | **TD** |  |  |  |  |  |
|  |  | Time Effect | 18.63 | 4,100 | **0.43** | **>100** | **<0.001** |
| Math verification task | **ACC** | **Trained** |  |  |  |  |  |
|  |  | Time Effect | 9.09 | 1,61 | **0.13** | **8.11** | **0.004** |
|  |  | Group Effect | 1.60 | 1,61 | 0.03 | 0.59 | 0.210 |
|  |  | Interaction | 0.48 | 1,61 | 0.01 | 0.31 | 0.492 |
|  |  | **Untrained** |  |  |  |  |  |
|  |  | Time Effect | 1.31 | 1,61 | 0.02 | 0.29 | 0.257 |
|  |  | Group Effect | 0.57 | 1,61 | <0.01 | 0.40 | 0.453 |
|  |  | Interaction | 1.90 | 1,61 | 0.03 | 0.56 | 0.173 |
| Math production task | **RT** | **Trained** |  |  |  |  |  |
|  |  | Time Effect | 148.13 | 1,59 | **0.72** | **>100** | **< 0.001** |
|  |  | Group Effect | 2.87 | 1,59 | 0.05 | 0.87 | 0.096 |
|  |  | Interaction | 1.65 | 1,59 | 0.03 | 0.46 | 0.204 |
|  |  | **Untrained** |  |  |  |  |  |
|  |  | Time Effect | 13.72 | 1,59 | **0.19** | **61.54** | **<0.001** |
|  |  | Group Effect | 2.20 | 1,59 | 0.04 | 0.85 | 0.144 |
|  |  | Interaction | 6.06 | 1,59 | **0.09** | **3.59** | **0.017** |
