# Supplementary material for: Atypical cognitive training-induced learning and brain plasticity and their relation to insistence on sameness in children with autism
Source: eLife. 2023 Aug 3;12:e86035. doi: 10.7554/eLife.86035 (PMC10550286; doi:10.7554/eLife.86035)
Supplement: Supplementary file 4. [file elife-86035-supp4.docx]

**Supplementary** **File 4**

**Table 4:** Results of *t*-tests for behavioral performance

| Task | Measure | Contrast | Mean ± SD | | *t* | df | Cohen’s *d* | BF | *p* |
| --- | --- | --- | --- | --- | --- | --- | --- | --- | --- |
| Training  task | **IES** | **ASD vs. TD** | ASD | TD |  |  |  |  |  |
|  |  | Session 1 | 7.58±4.32 | 7.12±3.84 | 0.41 | 53 | 0.11 | 0.46^a^ | 0.68 |
|  |  | Session 2 | 5.58±2.83 | 5.70±2.65 | -0.16 | 53 | -0.04 | 0.29 | 0.87 |
|  |  | Session 3 | 5.42±3.86 | 4.98±2.13 | 0.52 | 53 | 0.14 | 0.30 | 0.61 |
|  |  | Session 4 | 4.51±2.62 | 4.55±2.03 | -0.06 | 53 | -0.02 | 0.29 | 0.95 |
|  |  | Session 5 | 3.86±1.47 | 4.25±1.99 | -0.84 | 53 | -0.23 | 0.28 | 0.41 |
|  | **Learning**  **rate** | **ASD vs. TD** | ASD | TD |  |  |  |  |  |
|  |  |  | -0.85±0.72 | -0.69±0.67 | -0.86 | 53 | -0.23 | 0.27 | 0.391 |
| Math  verification  task | **ACC**  **-trained** | **Pre vs. Post** | Pre | Post |  |  |  |  |  |
|  |  | ASD | 0.86±0.11 | 0.88±0.10 | -1.72 | 34 | -0.29 | 0.68^a^ | 0.095 |
|  |  | TD | 0.88±0.11 | 0.92±0.06 | -2.54 | 27 | **-0.48** | **2.91^a^** | **0.017** |
|  |  | **ASD vs. TD** | ASD | TD |  |  |  |  |  |
|  |  | Pre | 0.86±0.11 | 0.88±0.11 | -0.73 | 61 | -0.19 | 0.32 | 0.467 |
|  |  | Post | 0.88±0.10 | 0.92±0.06 | -1.67 | 61 | -0.42 | 0.83 | 0.101 |
|  | **ACC gain**  **-trained** | **ASD vs. TD** | ASD | TD |  |  |  |  |  |
|  |  |  | 0.04±0.11 | 0.06±0.12 | -0.73 | 61 | -0.18 | 0.32 | 0.470 |
|  | **ACC**  **-untrained** | **Pre vs. Post** | Pre | Post |  |  |  |  |  |
|  |  | ASD | 0.84±0.12 | 0.84±0.13 | -0.17 | 34 | -0.03 | 0.74^a^ | 0.864 |
|  |  | TD | 0.88±0.11 | 0.84±0.13 | 1.73 | 27 | 0.33 | 0.18 | 0.095 |
|  |  | **ASD vs. TD** | ASD | TD |  |  |  |  |  |
|  |  | Pre | 0.84±0.12 | 0.88±0.11 | -1.40 | 61 | -0.35 | 0.59^a^ | 0.168 |
|  |  | Post | 0.84±0.13 | 0.84±0.13 | -0.02 | 61 | 0.00 | 0.26 | 0.988 |
|  | **ACC gain** | **ASD vs. TD** | ASD | TD |  |  |  |  |  |
|  | **-untrained** |  | 0.02±0.16 | -0.04±0.14 | 1.35 | 61 | 0.34 | 0.55^a^ | 0.183 |
| Math  production  task | **RT (s)**  **-trained** | **Pre vs. Post** | Pre | Post |  |  |  |  |  |
|  |  | ASD | 6.53±2.89 | 3.85±2.21 | 8.68 | 32 | **1.51** | **>100** | **<0.001** |
|  |  | TD | 5.31±2.28 | 3.15±1.69 | 9.31 | 27 | **1.76** | **>100** | **<0.001** |
|  |  | **ASD vs. TD** | ASD | TD |  |  |  |  |  |
|  |  | Pre | 6.53±2.89 | 5.31±2.28 | 1.80 | 59 | 0.46 | 1.00 | 0.078 |
|  |  | Post | 3.85±2.21 | 3.15±1.69 | 1.37 | 59 | 0.35 | 0.57^a^ | 0.178 |
|  | **RT gain** | **ASD vs. TD** | ASD | TD |  |  |  |  |  |
|  | **-trained** |  | -0.41±0.19 | -0.41±0.16 | -0.03 | 59 | -0.01 | 0.28 | 0.979 |
|  | **RT (s)**  **-untrained** | **Pre vs. Post** | Pre | Post |  |  |  |  |  |
|  |  | ASD | 6.77±2.91 | 5.47±2.91 | 3.70 | 32 | **0.64** | **38.68** | **<0.001** |
|  |  | TD | 5.26±2.40 | 5.00±2.59 | 1.35 | 27 | 0.26 | 0.46^a^ | 0.187 |
|  |  | **ASD vs. TD** | ASD | TD |  |  |  |  |  |
|  |  | Pre | 6.77±2.91 | 5.26±2.40 | 2.19 | 59 | **0.56** | **1.88^a^** | **0.033** |
|  |  | Post | 5.47±2.91 | 5.00±2.59 | 0.66 | 59 | 0.17 | 0.31 | 0.512 |
|  | **RT gain** | **ASD vs. TD** | ASD | TD |  |  |  |  |  |
|  | **-untrained** |  | -0.19±0.24 | -0.06±0.19 | -2.23 | 59 | **-0.57** | **1.57^a^** | **0.029** |

**^a^** insufficient evidence for H0 or H1
