# Supplementary material for: Atypical cognitive training-induced learning and brain plasticity and their relation to insistence on sameness in children with autism
Source: eLife. 2023 Aug 3;12:e86035. doi: 10.7554/eLife.86035 (PMC10550286; doi:10.7554/eLife.86035)
Supplement: Supplementary file 5. [file elife-86035-supp5.docx]

**Supplementary** **File 5**

**Table 5:** Results of chi-squared tests for dominant strategy use

|  |  |  |  | Memory-based | Rule-based | $\boldsymbol{\chi}^{\boldsymbol{2}}$ | *df* | $\boldsymbol{\phi}$ | BF | *p* |
| --- | --- | --- | --- | --- | --- | --- | --- | --- | --- | --- |
| Dominant  strategy use | **Group vs. Strategy**  **(trained)** | **Pre** | **ASD** | 4 | 29 | 0.40 | 1 | 0.08 | 0.50 | 0.312 |
|  |  |  | **TD** | 5 | 23 |  |  |  |  |  |
|  |  | **Post** | **ASD** | 17 | 16 | 4.81 | 1 | **0.28** | **3.39** | **0.028** |
|  |  |  | **TD** | 22 | 6 |  |  |  |  |  |
|  | **Group vs. Strategy**  **(untrained)** | **Pre** | **ASD** | 4 | 29 | 0.03 | 1 | 0.02 | 0.46 | 0.864 |
|  |  |  | **TD** | 3 | 25 |  |  |  |  |  |
|  |  | **Post** | **ASD** | 11 | 22 | 0.58 | 1 | 0.10 | 0.42 | 0.444 |
|  |  |  | **TD** | 12 | 16 |  |  |  |  |  |
| Strategy differentiation between trained and untrained problems | **Problem vs. Strategy**  **(ASD)** | **Pre** | **Trained** | 4 | 29 | 0 | 1 | 0.00 | 0.43 | 1.000 |
|  |  |  | **Untrained** | 4 | 29 |  |  |  |  |  |
|  |  | **Post** | **Trained** | 17 | 16 | 2.23 | 1 | 0.18 | 0.90 | 0.135 |
|  |  |  | **Untrained** | 11 | 22 |  |  |  |  |  |
|  | **Problem vs. Strategy**  **(TD)** | **Pre** | **Trained** | 5 | 23 | 0.58 | 1 | 0.10 | 0.57 | 0.445 |
|  |  |  | **Untrained** | 3 | 25 |  |  |  |  |  |
|  |  | **Post** | **Trained** | 22 | 6 | 7.49 | 1 | **0.37** | **13.24** | **0.006** |
|  |  |  | **Untrained** | 12 | 16 |  |  |  |  |  |
