# Supplementary material for: Atypical cognitive training-induced learning and brain plasticity and their relation to insistence on sameness in children with autism
Source: eLife. 2023 Aug 3;12:e86035. doi: 10.7554/eLife.86035 (PMC10550286; doi:10.7554/eLife.86035)
Supplement: Supplementary file 6. [file elife-86035-supp6.docx]

**Supplementary File 6**

**Table 6:** Brain regions showing significant group by behavior interaction on neural representational plasticity between pre- and post-training for trained problems

| Region | MNI coordinates | | | Peak  (*F*) | Cluster size  (voxel) | Cohen’s *f* ^2^ |
| --- | --- | --- | --- | --- | --- | --- |
|  | **x** | **y** | **z** |  |  |  |
| R MTL/PHG | 30 | -48 | 2 | 28.43 | 211 | 0.69 |
| L MTL/PHG | -16 | -36 | -14 | 23.97 | 108 | 0.58 |
| R FEF/preCG | 28 | -20 | 60 | 21.27 | 209 | 0.52 |
| R IPS/LOC | 30 | -64 | 50 | 20.54 | 116 | 0.50 |
| R LOC | 30 | -78 | 16 | 19.59 | 76 | 0.48 |
| R MFG/FP | 28 | 38 | 28 | 18.55 | 95 | 0.45 |

These regions showed significant group by behavior interaction on NRP for trained problems at the threshold of *p* < 0.005 height and *p* < 0.05 cluster extent, GRF corrected. The learning gains, computed as changes in accuracy for trained problems in the verification task during the fMRI scan, was used as behavioral measure in this analysis. L, Left; R, Right; MTL, medial temporal lope; PHG, Parahippocampal gyrus; FEF, Frontal eye fields; preCG, Precentral gyrus; IPS, Intraparietal sulcus; LOC, Lateral occipital cortex; MFG, Middle frontal gyrus; FP, Frontal pole; NRP, neural representational plasticity.
