# Supplementary material for: Atypical cognitive training-induced learning and brain plasticity and their relation to insistence on sameness in children with autism
Source: eLife. 2023 Aug 3;12:e86035. doi: 10.7554/eLife.86035 (PMC10550286; doi:10.7554/eLife.86035)
Supplement: Supplementary file 7. [file elife-86035-supp7.docx]

**Supplementary File 7**

**Table 7**: Results of brain-behavior association between region of interest (ROI)-based neural representational plasticity and learning gains.

| Region | *F* | df | Cohen’s *f* ^2^ | *p* |
| --- | --- | --- | --- | --- |
| L MTL | 8.15 | 1,41 | **0.20** | **0.007** |
| R MTL | 8.87 | 1,41 | **0.22** | **0.005** |
| L IPS | 8.08 | 1,41 | **0.20** | **0.007** |
| R IPS | 0.06 | 1,41 | <0.01 | 0.805 |

The group by behavior interaction effects on NRP for trained problems at the threshold of *p* < 0.05 FDR corrected. The learning gains was computed as changes in accuracy for trained problems in the verification task during the fMRI scan. L, Left; R, Right; MTL, Medial temporal lope; IPS, Intraparietal sulcus; NRP, Neural representational plasticity.
