# Supplementary material for: Atypical cognitive training-induced learning and brain plasticity and their relation to insistence on sameness in children with autism
Source: eLife. 2023 Aug 3;12:e86035. doi: 10.7554/eLife.86035 (PMC10550286; doi:10.7554/eLife.86035)
Supplement: Supplementary file 8. [file elife-86035-supp8.docx]

**Supplementary File 8**

**Table 8**: Moderation results for RRIB sub-scores on the association between brain and behavioral measures

|  | RRIB sub-scores | Interaction | | | |  | Model | | |
| --- | --- | --- | --- | --- | --- | --- | --- | --- | --- |
|  |  | ***b*** | ***se*** | ***t*** | ***p*** |  | ***R*^2^** | ***F*** | ***p*** |
| NRP (R MTL)  - learning gains | Insistence on sameness | -0.09 | 0.29 | -0.32 | 0.755 |  | 0.33 | 2.50 | 0.099 |
|  | Circumscribed interests | 0.36 | 0.32 | 1.12 | 0.281 |  | 0.28 | 1.97 | 0.162 |
|  | Repetitive motor behavior | -0.48 | 0.38 | -1.28 | 0.221 |  | 0.34 | 2.60 | 0.091 |
| NRP (L MTL)  - learning gains | Insistence on sameness | **-0.85** | **0.31** | **-2.76** | **0.015** |  | **0.53** | **5.64** | **0.009** |
|  | Circumscribed interests | -0.21 | 0.26 | -0.80 | 0.437 |  | 0.33 | 2.51 | 0.098 |
|  | Repetitive motor behavior | 0.04 | 0.40 | 0.11 | 0.916 |  | 0.31 | 2.25 | 0.125 |
| NRP (R IPS)  - learning gains | Insistence on sameness | **-0.44** | **0.19** | **-2.28** | **0.038** |  | **0.34** | **4.09** | **0.026** |
|  | Circumscribed interests | 0.01 | 0.32 | 0.03 | 0.975 |  | 0.07 | 1.46 | 0.264 |
|  | Repetitive motor behavior | -0.09 | 0.28 | -0.34 | 0.738 |  | 0.08 | 1.52 | 0.250 |

RRIB, Repetitive and restricted interests and behaviors; NRP, Neural representational plasticity; MTL, Medial temporal lope; IPS, Intraparietal sulcus.
